# Mental health difficulties, coping mechanisms and support systems among school-going adolescents in Ghana: A mixed-methods study

**DOI:** 10.1371/journal.pone.0250424

**Published:** 2021-04-22

**Authors:** Noella Dufie Addy, Faith Agbozo, Silvia Runge-Ranzinger, Pauline Grys

**Affiliations:** 1 Heidelberg Institute of Global Health, Heidelberg University Medical Faculty, Mannheim, Germany; 2 Department of Family and Community Health, School of Public Health, University of Health and Allied Sciences, Ho, Ghana; University of Cape Coast, GHANA

## Abstract

**Background:**

Although adolescents are highly vulnerable to mental health challenges, they receive little attention, especially in developing countries. We investigated the mental health difficulties (MHDs) faced by adolescent students in four senior high schools in Ghana, their coping strategies and support systems.

**Methods:**

In this convergent mixed-methods study, quantitative data was obtained using validated strengths and difficulties questionnaire to assess the mental health of 405 adolescents. Qualitative data was collected through in-depth interview with 18 teachers and seven focused group discussions with 35 students. Adjusted odds ratios (OR) for MHDs were estimated through ordinal logistic regression in Stata 14.2. Qualitative data was analyzed inductively and deductively using ATLAS.ti 7.1.

**Results:**

Over half (58.5%) experienced peer (20.5%), emotional (16.3%), conduct (13.3%) and hyperactivity (3.0%) problems, whereas 5.4% exhibited prosocial behaviours. MHDs were associated with females (OR = 2.27, 95% CI: 1.47–3.50), bullying (OR = 1.72, CI: 1.07–2.75), domestic violence (OR = 1.87, CI: 1.10–3.17), substance abuse (OR = 8.14, CI: 1.41–46.8), academic pressure (OR = 2.40, CI: 1.30–4.42) and self-perceived poor school performance (OR = 7.36 CI: 2.83–19.16). Qualitatively, we identified financial challenges, spiritual influences, intimate relationships, bullying, and domestic violence as the main themes attributed to MHDs. Coping strategies included isolation, substance use and spiritual help. The main school-based support system was the guidance and counselling unit, but there were complaints of it been ineffective due to trust and confidentially issues, and non-engagement of qualified counsellors.

**Conclusions:**

As many triggers of MHDs emanate in schools, we need mental health-friendly school environments where trained psychotherapists head counselling centres. We propose incorporating mental health education into school curricula and generating surveillance data on adolescent’s mental health.

## Introduction

As mental health gains global priority and centrality to health and development agendas, efforts have intensified to focus on the mental health of adolescents (typically defined as persons in their second decade of life [10–19 years]). This is imperative as adolescence is characterized by extensive physical and social development whereby capabilities essential for successful progression into adulthood are established [1]. Fifty percent of mental health disorders that occur during adolescence, when unaddressed, can progress into adulthood [2] and have a lasting effect on personality development, educational achievement, psychosocial relationships, work productivity, physical health and disability [2, 3].

Globally,10–20% of adolescents experience mental health disorders [4]. Anxiety and depression is among the leading causes of illness and disability, particularly among adolescent girls [5]. In sub-Sahara Africa where adolescents and young adults constitute 30–35% of the population [6], 14% suffer from psychological distress [7]. In Ghana, 19% of the population experience moderate or severe depression [8]. Even among tertiary students, 8% suffer severe depression [9]. No wonder suicidal tendencies and self-harm are common in low- and middle-income countries (LMICs) [5, 10] and is rated as the fifth top cause of death [11, 12] among persons aged 10 to 24 years [13]. Yet, mental health needs of adolescents have generally been neglected [14, 15].

Among school-going adolescents, the school environment could potentially contribute to mental health promotion and prevention, or adversely affect the mental health and wellbeing of students [13, 16]. Behaviours typical of the school and classroom settings such as bullying, teasing, difficulty adjusting to rules, poor relationship with teachers and peers, academic pressure, examination phobia, unhealthy competitions, peer pressure, peer victimization, peer marginalization and repeated oppression [17–19] can put adolescents on edge. The consequence is inappropriate behaviours including rebellion against authority, use of alcohol, tobacco and illicit drugs, and risky sexual behaviours [4, 5]. Aside triggering mental disorders, these behaviours can lead to teenage pregnancy, sexually transmitted infections, low educational achievement, and even school dropout [13].

However, some triggers of mental health disorders are subtle but deep-rooted within the cultural, psychosocial, economic, family and community systems. Poor relationship with parents and siblings, high expectations from parents, family conflicts, parental neglect, single parenthood, homelessness, child abuse, poverty, parental mental illness, living in vulnerable settings and lack of support for adolescents with chronic conditions and disabilities are classical in both high and LMICs [15, 20–23].

In school, students adopt an array of mechanisms to deal with mental health issues varying from religious support, positive reinterpretation, active coping, planning, and use of instrumental support [18]. Other strategies include breathing exercises, regular visits to a counsellor, talking to someone, temporary distraction, social networking, frequent exercising, healthy eating, meditation, joining a club, mindfulness, to calming strategies [24]. While some adolescents show resilience and resourcefulness in adapting effective coping strategies, approaches such as disengagement, isolation, over-indulgence, grieving and internalized coping strategies [25, 26] often pose negative consequences to the adolescent’s physical health while also aggravating existing mental health conditions [18, 26, 27].

Despite the role of optimum mental health on the overall quality of life, community, family and school set-ups are often not enabling enough to promote the social, emotional, psychological skills and lifestyle habits of adolescents [28]. Largely, mental health is on the periphery of health policy and developmental agenda as strategies to promote enabling and nurturing environments for optimal mental health is still lacking. This is evident by the scarcity of epidemiological and large-scale data on the magnitude of adolescents’ mental health problems, and coping strategies. Stigmatization of mental illness, coupled with lack of innovative psychosocial interventions and counselling therapy, as well as limited human and technical capacity to deliver mental health services is contributing to the hesitancy of patients in seeking help [5, 28].

Studies relating to the mental health of school-going adolescents and youths enrolled in basic, second cycle and tertiary institutions in Ghana abound. Herein, issues including bullying and victimisation, abuse of alcohol and illicit drugs, as well as academic stress, depression, suicidal behaviours and gender dynamics have received much research attention in recent times [29–33]. However, many of these studies are concentrated among adolescents in urban centres. Also, few studies have triangulated the findings using a mixed-methods design while data is limited to interventions currently available to support school-going adolescents. Recognizing the spectrum of mental health issues encountered by adolescents and the support services in place will guide to make the school environment more mental health-friendly, facilitate early identification of risk factors for future mental illness and inform the most suitable support services that will mitigate mental health difficulties [28].

It is on these bases that we conducted this mixed-method study among adolescents enrolled in second cycle schools (10^th^ to 12^th^ grade) in rural and peri-urban areas in Ghana to investigate the mental health issues experienced, coping strategies adopted and the support systems offered to help deal with the problems. The specific objective for the quantitative study was to assess the types and prevalence of self-perceived mental health problems experienced by the adolescents on the hypothesis that mental health problems are under-estimated among school-going adolescents in rural and peri-urban areas in Ghana. To gain more insights on the quantitative results, we sought to answer three qualitative research questions: (1) what are the triggers for mental health difficulties among school-going adolescents?; (2) how do they adapt to these difficulties?; and (3) which support services are provided as part of the school system?

## Materials and methods

### Mixed methods design

**Fig 1:**
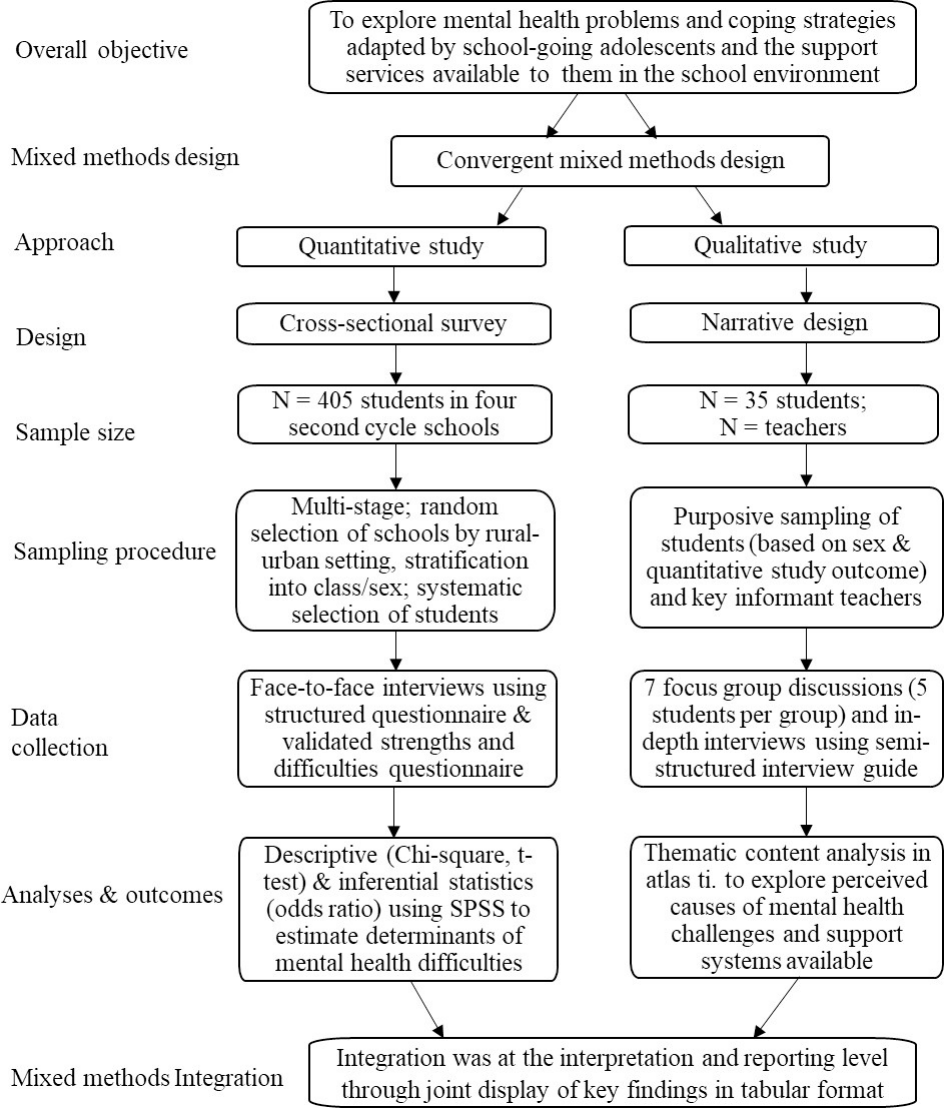
The convergent mixed-methods procedure used in this study.

An overview of the methodology employed is illustrated in Fig 1. The convergence triangulation mixed-methods model was used. Both quantitative and qualitative data were collected in parallel, but the analysis was done separately and the results were integrated by merging at the reporting level through a joint display of key findings [34]. As this design enhances the ease of comparing and contrasting quantitative results with qualitative findings [34], we could validate and interpret the key findings. The quantitative design was a cross-sectional survey while the qualitative part was a descriptive narrative study.

### Study setting

This study was conducted in four senior high schools (SHS) in the Techiman Municipality of the newly created Bono-East Region located in the middle belt of Ghana. According to the structure of the educational system in Ghana, SHS are second cycle institutions spanning the 10^th^ to the 12^th^ grade of formal education. All the eight public SHS in the municipality were stratified into rural and urban geographical locations. Two schools were randomly selected from each stratum. Per the Ghana Statistical Service criteria, localities with 5,000 or more and less than 5,000 inhabitants were classified as urban and rural, respectively [35]. We also considered infrastructural development of each school as well as the availability of health facilities, transportation network and commercial activities in the area where a school was situated.

### Procedures

#### Sample size and sampling

The sample size for the quantitative part was determined based on the total population of 2,791 students in the four study institutions. A 95% confidence interval corresponding to a 5% error margin, a reliability coefficient of 1.96 and a 50% response distribution was used as the magnitude of the problem is unknown in the study setting, yielding a sample size of 384. The target population comprised all day (non-residential) and boarding (residential) students in the four institutions.

Sampling was multi-stage. In each school, participants were stratified according to their class (grade). We used systematic sampling to select a proportionate number of males and females from each class. The qualitative study had 53 participants comprising 35 students and 18 teachers (Fig 1). Inclusion of teachers was on the premise that they might be better informed on the support systems and services institutionalized to promote the mental health of adolescents within the school setting.

In each school, four to five teachers and ten students (five males and five females) were interviewed except in one school where male students were unavailable. Eligibility criteria for the teachers were holding an administrative position in the school and attaining at least four years experience of working with students. The teachers interviewed included four assistant headmasters, two senior housemasters, four senior house mistresses, four guidance and counselling coordinators and four class tutors. Regarding the students, those who participated in the cross-sectional survey and provided insightful responses were eligible for selection into the qualitative study.

#### Data collection

Data was collected in 2017, between April and June. Participants completed a structured self-designed questionnaire administered via face-to-face interviews. The questionnaires were distributed by hand to the students during school hours and they were given some few hours to complete them. The lead researcher was present to clarify any questions. The self-administered questionnaire elicited information on participants’ socio-demographic characteristics and sources of mental health problems. For the qualitative data, in-depth interviews and focus-group discussions (FGDs) were conducted with the teachers and students, respectively, using two different semi-structured interview guides. The interview guide had three broad questions covering triggers of mental health problems among students, coping mechanisms and school-based interventions. All interviews were conducted in English and lasted between 30–45 minutes. Seven FGDs were conducted comprising of five participants per group. As theoretical saturation was reached, the interviews were terminated. To ensure privacy and confidentiality, the questionnaire was devoid of identifiable information on the participants and that also included the name of the school. The questionnaire was coded, and a separate list of code-to-name was kept to aid in matching-up. Participation was completely voluntary, and only participants who consented had their audio recorded.

### Measures

Using the questionnaire, we measured key socio-demographic indicators including age, sex, family size, parents’ occupation, and student’s residential status (day vs boarding student). Experience of domestic violence andbullying, use of illicit drugs and alcohol, and psychosocial/financial support from parents were the triggers of mental health problems measured. The questions were close-ended with multiple response categories that indicated the frequency of occurrence of the event.

We assessed participants’ general mental health using the strengths and difficulties questionnaire (SDQ); a validated screening tool developed to examine mental health among children and young people [36]. SDQ has been successfully used in previous studies in Ghana [9, 37]. We employed the self-reported version of the SDQ used for children and young persons. The questionnaire which assesses the total mental strengths and difficulties of an individual has 25 items grouped into five subscales of five items each that measure conduct, hyperactivity, emotional and peer relationship problems and prosocial behaviours. Example of questions from the SDQ and the mental health problem it measures is as follows: “I worry a lot” (emotional problems); “I fight a lot” (conduct problems); “I am restless” (hyperactivity problems); “I am usually on my own” (peer problems); and “I try to be nice to other people” (prosocial problems). Each subscale has five questions with three response categories ranging from not true (rated zero), somewhat true (rated one) to certainly true (rated two). Cumulative scores were calculated for each subscale and categorized as normal, borderline or abnormal (Table 2). Possible scores for the total strengths and difficulties ranged between 0–40 whereas the subscale was 0–10 [36]. Except for prosocial behaviours, higher scores were suggestive of abnormalities.

#### Reliability and rigour

Using the mixed methods design helped obtain different but complementary information that facilitated a deeper understanding of the mental health problems faced by school-going adolescents. It also aided the validation of the qualitative findings with the quantitative results. Based on the Cronbach alpha calculated, the reliability of the SDQ in measuring the total strengths and difficulties, as well as the emotional, hyperactivity, conduct, peer relationship and prosocial constructs, was acceptable (0.730). In terms of rigour of the qualitative study, we ensured credibility by piloting the test instrument; engaging field workers with required training and research skills; obtaining data from duo sources (in-depth interviews for the teachers and FGD for the students) and repeatedly probing for details or clarity during the interviews. Dependability was safeguarded by developing an audit trail where a detailed record of the data collection process was kept, alongside taking field notes, double coding and checking intercoder accuracy. Applying multiple triangulation techniques through the mixed methodology and varied data sources were useful in ensuring confirmability. We addressed transferability by purposefully recruiting participants from multiple study sites, and applying the concept of data saturation in determining the appropriate sample size for the qualitative part.

### Statistical analysis

Quantitative data were analyzed using STATA software (version 14.2). Descriptive data involving continuous and categorical data were summarized using t-test and Chi-square test, respectively. Using ordinal logistic regression, we estimated the adjusted odds ratio (AOR) for experiencing mental health difficulties; including emotional, hyperactivity, conduct and peer relationship problems; as well as exhibiting prosocial behaviours. The ordinal levels were normal, borderline and abnormal. The independent variables were age, sex, residential status, psychosocial and financial support, academic performance, exposure to domestic violence, alcohol and illicit drugs. Two-tailed with p-value <0.05 and any confidence interval (CI) excluding 1.0 were statistically significant.

Qualitative interviews were audio-recorded, transcribed verbatim and the transcriptions exported into Atlas-ti software (version 7.1) for analysis. Codes were generated a priori based on the research questions plus evidence from literature, as well as inductively as new concepts emerged. The codes were carefully examined for recurring themes. After relevant themes were mapped out, related themes were grouped, leading to the completion of the thematic analysis. Important themes with accompanying quotes were extracted and reported.

### Ethics

This study protocol was reviewed and approved by the Ethics Committee of the Heidelberg University Medical Faculty, Germany (S-192/2017). Administrative approval was obtained from the Techiman Municipal Education Office. The study protocol was explained to all potential participants emphasizing the purpose, the information elicited, voluntary participation and how their privacy and confidentiality would be maintained. All the teachers, and students aged 18 years and above provided their written informed consent. Similarly, students below 18 years signed the assent form signifying their willingness to participate. All non-residential students who provided assent to participate were given the participant information sheet to send home, and in turn, explain the study to their parents or legal guardians. Those who agreed that their wards participate provided written informed consent in the form of signatures or thumb-print on the information sheet. In the case of boarding students, consent was obtained from senior housemasters and mistresses who are the legal guardians of residential students. Participation of minors was conditional on receiving both the written informed consent and assent forms.

## Results

### Socio-demographic characteristics of participants

Overall, 405 students were recruited. No student declined to participate nor withdrew the consent from the survey. However, five students declined to participate in the FGD without giving any reason. Also, one student and two teachers requested for their audio not to be recorded. We had 67.7% (n = 274) and 32.3% (n = 131) students attending schools in urban and rural areas respectively. In Table 1, we present their socio-demographic characteristics. The proportion of females (44.9%) to males (53.9%) was statistically similar. The age of the students ranged from 12–20 years (mean 17.6±1.35). Comparing students enrolled in urban to rural schools, we found a significant difference in their family size, residential status, academic performance, parents’ occupation and financial capabilities, experience with bullying and alcohol intake.

**Table 1:** Socio-demographic characteristics of study participants stratified according to the geographical location of the school.

Characteristics	Total (n = 405)	Urban schools (n = 274)	Rural schools (n = 131)	P-value^a^
Mean number of siblings^b^	5.32 ± 2.695	5.26 ± 2.57	5.44 ± 2.52	0.275
Mean age (years)	17.68 ±1.340	17.69 ±1.17	17.66 ±1.63	0.132
<17 years (%)	199(49.1)	132(48.4)	67(50.8)	0.672
≥18 years (%)	206(50.9)	141(51.6)	65(49.2)	
*Sex (%)*				0.372
Male	221 (54.6)	151 (55.3)	70 (53.0)	
Female	184 (45.4)	123 (44.7)	61 (47.0)	
*Residential Status (%)*				***<0*.*0001***
Day student	202 (49.9)	99 (37.7)	103 (75.0)	
Boarding student	203 (50.1)	170 (62.3)	33 (25.0)	
*Religion affiliation (%)*				0.712
Catholic	82 (20.2)	57 (20.9)	25 (18.9)	
Islam	63 (15.6)	43 (15.8)	20 (15.2)	
Pentecostal	99 (24.4)	62 (22.7)	37 (28.0)	
Other denominations	161 (39.8)	111 (40.7)	50 (37.9)	
*Mother’s occupation (%)*				***0*.*037***
Unemployed	9 (2.2)	7 (2.6)	2 (1.5)	
Formal sector	30 (7.4)	26 (9.5)	4 (3.0)	
Informal sector	366 (90.4)	240 (87.9)	126 (95.5)	
*Father’s occupation (%)*				***0*.*014***
Unemployed	9(2.2)	8(2.9)	1(0.8)	
Formal sector	59(14.6)	48(17.	11(8.3)	
Informal sector	337(83.2)	217(79.5)6)	120(90.9)	
*Academic performance*^*c*^^¶^ *(%)*				***<0*.*0001***
Excellent	65 (16.0)	49 (17.9)	16 (12.1)	
Very good	139 (34.3)	78 (28.6)	61 (46.2)	
Good	152 (37.5)	101 (37.0)	51 (38.6)	
Fair ^¶^	47 (11.6)	43 (15.8)	4 (3.0)	
Repeater	2 (0.5)	2 (0.7)	0 (0.0)	
*Domestic violence (%)*				0.511
Yes	94(23.2)	63(23.1)	31(23.5)	
No	311(76.8)	210(76.9)	101(76.5)	
*Parents’ finance support (%)*				***0*.*001***
Yes, until end of SHS	175(43.3)	106(39.0)	69(52.3)	
Yes, to university	134(33.2)	106(39.0)	28(21.2)	
Not sure	95(23.5)	60(22.1)	35(26.5)	
*Bullied in school (%)*				***0*.*001***
Yes, during JHS	58(14.4)	25(9.2)	33(25.2)	
Yes, at SHS	216(53.6)	151(55.5)	65(49.6)	
No, never	129(32.0)	96(35.3)	33(25.2)	
*Intake of alcohol (%)*				***0*.*011***
Yes	33 (8.1)	29 (10.6)	4 (3.0)	
None at all	372(91.9)	244 (89.4)	128 (97.0)	
*Use of illicit drugs*				0.597
Yes	16 (4.0)	12 (4.4)	4 (3.0)	
None at all	389 (96.0)	261 (95.6)	128 (97.0)	

^a^ T-test was used for continuous variables and Fisher’s exact test for categorical variables.

^b^ Siblings includes the participant.

^c^ Academic performance was based on the students’ self-perceived assessment.

^¶^ Multiple comparisons involved post hoc analysis using Bonferroni adjustment.

JHS implies Junior High School (9^th^ grade); SHS implies Senior High School (12^th^ grade).

### Mental health strengths and difficulties

From a possible score range of 0–40, the mean strengths and difficulty score was 20.50 (SD = 5.54) (Table 2). About half (53.6%) of the students fell within the “abnormal” classification suggesting a higher likelihood for clinical mental health problems. Conversely, a quarter (26.9%) were “borderline” suggesting a pre-clinical range of mental health problems. Peer problems were the most common, affecting 20%, followed by emotional (16.3%), conduct (13.3%) and hyperactivity problems (3.0%). Prosocial behaviours, a mental health strength, was exhibited by only 5.4% of the students.

**Table 2:** Mean scores and distribution of mental health strengths and difficulties.

Mental health traits	Scoring ^a^ & results	Mean score (SD)	Range	Normal n (%)	Borderline n (%)	Abnormal n (%)
Emotional symptoms	Scoring	-	0–10	0–5	6	7–10
	Results	3.87 (2.48)	0–10	307 (75.8)	32 (7.9)	66 (16.3)
Conduct problems	Scoring	-	0–10	0–3	4	5–10
	Results	2.20 (2.01)	0–10	310 (76.5)	41 (10.1)	54 (13.3)
Hyperactivity	Scoring	-	0–10	0–5	6	7–10
	Results	2.55 (1.85)	0–8	375 (92.6)	18 (4.4)	12 (3.0)
Peer problems	Scoring		7–10	0–3	4–5	7–10
	Results	4.01 (1.82)	1–9	178 (44.0)	144 (35.6)	83 (20.5)
Prosocial behaviours	Scoring		0–10	6–10	5	0–4
	Results	7.87 (1.96)	1–10	351 (86.7)	32 (7.9)	22 (5.4)
Total difficulty score	Scoring		0–40	0–15	16–19	20–40
	Results	20.50 (5.54)	8–36	79 (19.5)	109 (26.9)	217 (53.6)

^a^ The range of scores specified in the strengths and difficulty questionnaire was applied [36].

SD, standard deviation; n, number of observations

**Fig 2:**
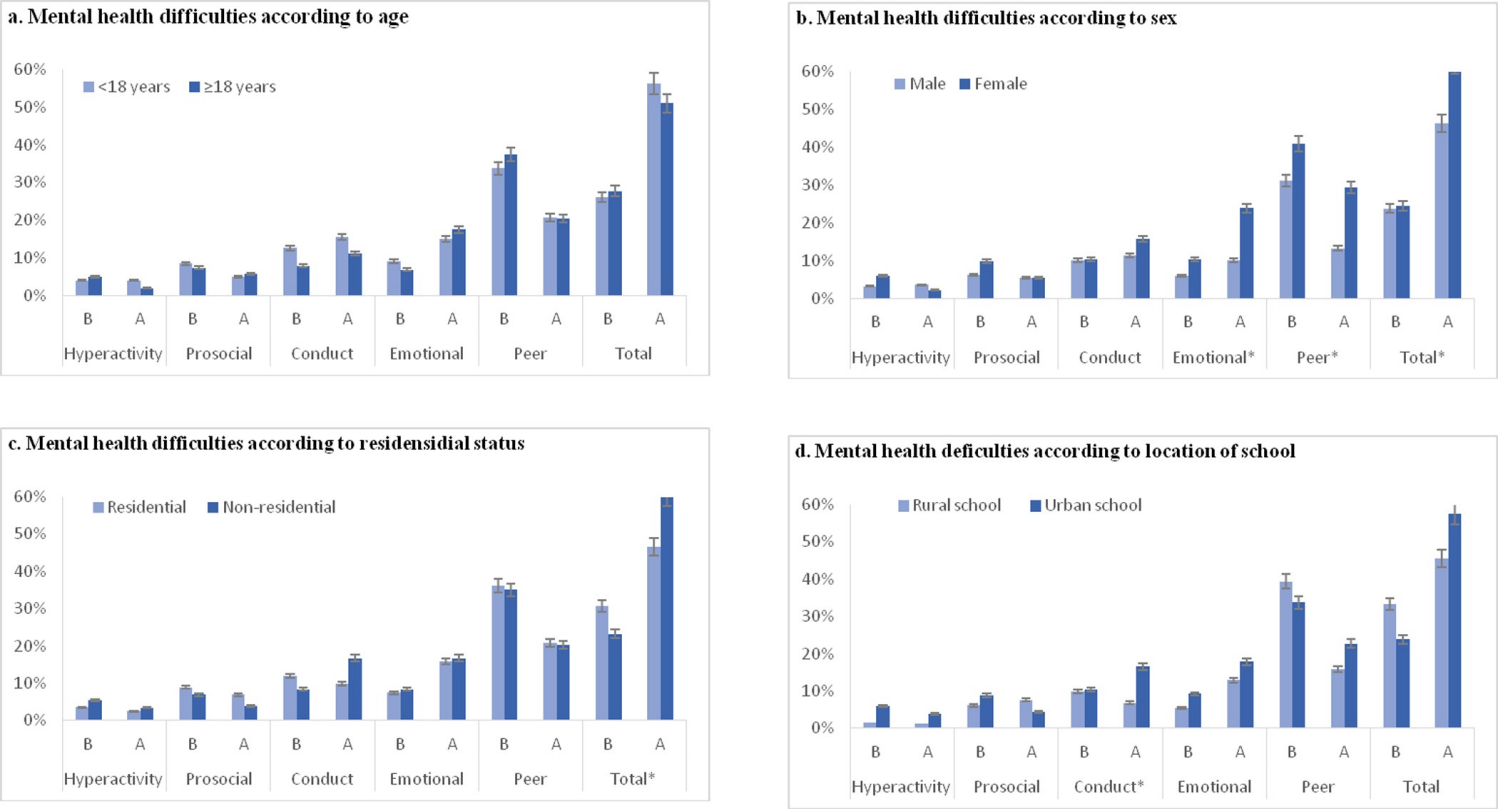
Stratification of mental health strengths and difficulties according to (a) age, (b) sex, (c) residential status and (d) location of the school. Footnote: * x^2^ < 0.005. B implies borderline and A implies abnormal mental health status.

Fig 2 shows the stratification of mental health strengths and difficulties according to age, sex, residential status and location of the school. Overall, more females reported mental health difficulties (62.5%) compared to males (46.2%) (p<0.0001), so were non-residential students (60.6%) compared to residential students (46.5%). Concerning specific sub-scales, significant differences were found among males and females in terms of emotional symptoms (10.0% vs 23.9, p<0.0001) and peer problems (13.19% vs 29.3, p<0.0001). Students in urban schools (16.5%) had more conduct problems than their rural school counterparts (6.8%) (p = 0.019).

### Determinants of mental health difficulties

In Table 3 are the factors associated with experiencing mental health difficulties. Females had a two-fold likelihood of experiencing mental health difficulties (AOR = 2.27, 95% CI: 1.47–3.50). Other associated determinants were self-perceived poor academic performance in school (AOR = 7.36, 95% CI: 2.83–19.16), external pressure to excel in academic work (AOR = 2.40, 95% CI: 1.30–4.42), illicit drug use (AOR = 8.14, 95% CI: 1.41–46.8), difficulty accessing psychosocial support (AOR = 2.14, 95% CI: 1.39–3.30), conflict in the family (AOR = 1.87, 95% CI: 1.10–3.17) and peer bullying in school (AOR = 1.72, 95% CI: 1.07–2.75). Risk factors for experiencing conduct, hyperactivity, emotional and peer relationship problems as well as exhibiting prosocial behaviours are presented in Table 3.

**Table 3:** Ordinal logistic regression model showing the predictors for experiencing mental health difficulties and the subscales.

Explanatory variables	Categories	Total difficulties AOR (95% CI)	Emotional AOR (95% CI)	Conduct AOR (95% CI)	Hyperactivity AOR (95% CI)	Peer AOR (95% CI)	Prosocial AOR (95% CI)
Age	≥18 years	Ref	Ref	Ref	Ref	Ref	Ref
	<18 years	0.92 (0.60–1.41	0.70 (0.41–1.17)	1.54 (0.94–2.52)	1.34 (0.59–3.04)	0.67 (0.45–1.01)	0.95 (0.52–1.73)
Sex	Male	Ref	Ref	Ref	Ref	Ref	Ref
	Female	*2*.*27 (1*.*47–3*.50)*	*3*.*86 (2*.*23–6*.65)*	1.23 (0.74–2.04)	1.10 (0.45–2.69)	*3*.*00 (2*.*00–4*.50)*	1.01 (0.53–1.91)
Residential status	Non-residential	Ref	Ref	Ref	Ref	Ref	Ref
	Residential	1.32 (0.83–2.09)	0.86 (0.49–1.52)	0.83 (0.48–1.45)	0.79 (0.30–2.10)	0.70 (0.45–1.09)	*0*.*46 (0*.*22–0*.93)**
Location of school	Rural	Ref	Ref	Ref	Ref	Ref	Ref
	Urban	1.14 (0.70–1.85)	1.68 (0.90–3.14)	*1*.*92 (1*.*03–3*.57)*	2.98 (0.83–10.64)	1.18 (0.74–1.87)	1.10 (0.53–2.27)
Perceived academic performance	Excellent	Ref	Ref	Ref	Ref	Ref	Ref
	Very good	1.50 (0.81–2.09)	1.82 (0.79–4.14)	0.71 (0.33–1.52)	*0*.*08 (0*.00-0-84)**	1.12 (0.61–2.05)	0.72 (0.25–2.07)
	Good	1.68 (0.92–3.07)	1.34 (0.59–3.03)	0.79 (0.38–1.529	1.64 (0.51–5.219)	1.20 (0.67–2.159	1.66 (0.65–4.28)
	Poor	*7*.*36 (2*.*83–19*.16)*	*3*.*96 (1*.*54–10*.17)**	1.90 (0.79–4.45)	3.10 (0.76–12.55)	*2*.*33 (1*.*10–4*.96)*	1.37 (0.37–5.12
External pressure to excel	None	Ref	Ref	Ref	Ref	Ref	Ref
	Little	1.16 (0.59–2.15)	1.03 (0.37–2.86)	1.29 (0.49–3.42)	0.60 (0.15–2.31)	1.07 (0.53–2.15)	0.36 (0.12–1.02)
	Moderate	1.03 (0.5–2.11)	1.20 (0.41–3.46)	1.31 (0.46–3.68)	0.29 (0.45–1.89)	1.04 (0.49–2.17)	0.62 (0.19–1.94)
	Intense	*2*.*40 (1*.*30–4*.42)**	2.36 (0.98–5.70)	1.74 (0.74–4.10)	0.50 (0.14–1.73)	1.45 (0.78–2.69)	0.41 (0.16–1.01)
Alcohol intake	No	Ref	Ref	Ref	Ref	Ref	Ref
	Drinks alcohol	1.25 (0.49–3.15)	0.98 (0.40–2.37)	1.44 (0.61–3.39)	1.18 (0.33–4.18)	*2*.*54 (1*.*22–5*.29)**	*3*.*29 (1*.*30–9*.55)*
Illicit drugs	No	Ref	Ref	Ref	Ref	Ref	Ref
	Uses drugs	*8*.*14 (1*.*41–46*.8)**	*6*.*86 (2*.*18–21*.55)**	1.03 (0.27–3.39)	*5*.*54 (1*.*20–25*.52)**	1.61 (0.50–5.19)	0.31 (0.02–3.40)
Suicidal thoughts	No	Ref	Ref	Ref	Ref	Ref	Ref
	Happens	6.96 (0.79–60.8)	1.93 (0.48–7.74)	1.79 (0.41–7.85)	4.92 (0.71–33.71)	*6*.*15(1*.*68–22*.45)**	1.77 (0.18–17.22)
Domestic violence	No	Ref	Ref	Ref	Ref	Ref	Ref
	Exposed	1.*87 (1*.*10–3*.17)**	1.52 (0.83–2.13)	*1*.*89 (1*.*08–3*.28)**	1.86 (0.77–4.50)	1.30 (0.82–2.08)	0.69 (0.31–1.54)
Bullying in school	Never	Ref	Ref	Ref	Ref	Ref	Ref
	Bullied at SHS Bullied at JHS	*1*.*72 (1*.*07–2*.75)** 1.90 (0.94–3.81)	1.10 (0.60–2.10) 1.36 (0.59–3.09)	*2*.*32 (1*.*23–4*.36)** *3*.*38 (1*.*50–7*.60)**	1.03 (0.37–2.88) 2.90 (0.75–11.17)	*1*.*64 (1*.*04–2*.58)** *1*.*30 (0*.*67–2*.*51)*	1.64 (0.76–3.54) 2.09 (0.76–5.71)
Psychosocial support	Family	Ref	Ref	Ref	Ref	Ref	Ref
	Friends	*2*.*14 (1*.*39–3*.30)**	1.45 (0.84–2.50)	*1*.*80 (1*.*07–3*.04)**	*4*.*53 (1*.*52–13*.47)**	*1*.*87 (1*.*24–2*.83)**	1.59 (0.32–7.69)
	None	1.81 (0.75–4.33)	*3*.*39 (1*.*31–8*.79)**	0.65 (0.19–21)	1.42 (0.22–9.16)	1.16 (0.49–2.74)	1.32 (0.24–7.07)
Parent’s financial capability	Till tertiary	Ref	Ref	Ref	Ref	Ref	Ref
	Till SHS	0.99 (0.61–1.60)	1.20 (0.66–2.17)	0.87 (0.49–1.53)	0.50 (0.32–2.51)	1.12 (0.71–1.77)	0.92 (0.44–1.89)
	Not capable	1.21 (0.68–2.13)	1.77 (0.88–3.55)	1.48 (0.74–2.94)	2.36 (0.77–7.16)	*1*.*78 (1*.*03–3*.08)**	1.73 (0.82–3.64)
	Model summary	Prob > chi^2^ = 0.0000 Pseudo R^2^ = 0.1260	Prob >chi^2^ = 0.0000 Pseudo R^2^ = 0.1294	Prob > chi^2^ = 0.0009 Pseudo R^2^ = 0.0810	Prob > chi^2^ = 0.0004 Pseudo R^2^ = 0.1890	Prob > chi^2^ = 0.0000 Pseudo R^2^ = 0.0972	Prob > chi^2^ = 0.2679 Pseudo R^2^ = 0.0744

*P < 0.01,

** P < 0.05. Number of observations = 398

AOR, Adjusted Odds Ratio; CI, confidence interval; JHS implies Junior High School (9^th^ grade); SHS implies Senior High School (12^th^ grade)

### Triggers of mental health problems

Inquiring the triggers for mental health problems, five main themes emerged: financial challenges; spiritual influences; issues arising from intimate relationships with the opposite sex; bullying victimization; and experience of domestic violence.

Most students mentioned financial challenge as a cause of mental health difficulties. This arose from the neglect of parental responsibilities (especially fathers), parental unemployment, and the loss of a parent(s). As a result, some students were compelled to work to pay their fees and fend for themselves. The implications were absenteeism leading to poor academic performance, depression, alienation and suicidal tendencies.

*“when they sack you for fees ……and you think like*, *you are not a human being and something [inner voice] tells you that go and kill yourself cos ……they [school authorities] are maltreating you as if you are not a human being”*. ________Female student

However, the teachers thought that spiritual influences played a role. It was commonly attributed to witches in the family whose intent was to retrogress the student’s education and prospects. Involvement in occultic groups was believed to also be culpable.

*“Some of the boarders were caught engaging in occultism…*. *they have been chanting and calling spirits*. *When they do things contrary to what the spirits asked them*, *then some of them develop mental problems…*. *they start hallucinating*”. ________Guidance and counselling coordinator

Some teachers believed that the students’ psychological distress originated from intimate relationships with the opposite sex. They explained that most of the students are not matured to engage in intimate relationships but are often influenced by their peers. Hence, when the students encounter problems in the relationship, they become “broken-hearted”. It is compounded by their inability to confide in a trusted person or seek support as the society frowns on such relationships among adolescents.

*“I was in a relationship with my senior*. *I enjoyed the prestige that came with it*. *But he suddenly left me when the next batch of first-year students came*. *I was heartbroken and couldn’t eat or study for months*”. ________Female student

Most SHS in Ghana run the boarding system where discipline and close monitoring is the norm. The expectation is that the child will learn communal living and become well nurtured. But some teachers expressed concern that confinement in the boarding house was not conducive for certain students as their movement is restricted and they have to dress, behave and even eat in a prescribed way. This was problematic especially for students from rural communities.

*“I’m talking about when they move from let’s say a typical village ……and now among hundreds of people*. *All of a sudden*, *your freedoms are curtailed because …*‥ *you now do certain things in certain ways*. *If you are not able to adjust very fast*, *it means that you have a mental challenge”*. ________Senior housemaster

Bullying victimisation in the boarding house was another trigger mentioned. The students anticipated that going to the boarding school will be a relief from domestic problems only to realise that they were saddled with non-academic activities while the “seniors will not let them be”.

*“We thought boarding students get much time to study without wahala [troubles]*. *Now*, *we work more than we study in the boarding house like weeding*, *cleaning and scrubbing and the seniors too are bullying us”*. ________Male student

Domestic violence including maltreatment at home emerged as a contributory factor to the mental health difficulties the adolescents’ faced. One participant who did not want the voice recorded said he started taking drugs when his father died, and his mother remarried, and he was maltreated by the step-father. Some students also reported being beaten with belts and canes, insulted or starved by both their biological and step-parents.

*“When I go home for vacation and my parents have a quarrel…*‥ *they turn it on me*, *at times slapping my face*. *It doesn’t make me sleep and when I come back to school*, *I will be thinking of them [the experience]*. *I won’t have the peace to study”*. ________Male student

### Coping strategies

In dealing with the problems, isolation, use of illicit drugs and seeking spiritual help were the main coping strategies.

Due to the stigma associated with mental illness, isolation, seclusion and avoidance of social contacts were the primary adaptations.

*“When you let people know what you are going through*, *they will think you are mad*. *I just keep to myself”*. ________Female student

Some boys admitted taking illicit drugs and narcotics particularly cannabis and amphetamine when faced with mental health challenges. According to the students, it helped them “forgot their hardships”, “makes them feel high” (important) and facilitated their studies by “improving their retentive memory”.

*“……some of us have been taking tramor (tramadol) and others smoke wee (marijuana) and ataya (a stimulant) because we think that when you smoke it*, *you forget your problems*, *you don’t think of that anymore”*. ________Male student

Another coping strategy was seeking help from supernatural sources; prayer being the first action. Others confided in their friends, entertained themselves, cried or listened to music as a means of coping.

*“When you start hearing voices*, *it’s only the spiritualist like fetish priests who can help you*. *So I just go there for help”*. ________Male student

### School-based interventions to promote adolescents’ mental health

Guidance and counselling unit setup in SHS as a directive from the Ghana Education Service is the main support-system presently available to address adolescents’ mental health difficulties. A popular teacher who is liked by most students, a staff who is a pastor, pastor’s spouse or a church deacon is appointed to head the unit. Teachers who have training in counselling or psychology are usually also part of the team. However, the teachers lamented that the criteria for selecting the head of the unit should be based on subject-specific expertise and adequate training rather than popularity, association with or leadership in a religious group.

*“The teachers who are part of the counselling unit are the same people teaching these students*. *Because of that*, *the students are shy to go to them…even most of them do not have the required training to be part of the unit but because he is a pastor or something*.*”* ________Class tutor

The students on the other hand complained that the counsellors were judgmental and “preached” instead of providing support and providing alternate courses for action. They also complained that the services provided at the unit did not address their problems.

*“I know the school has a counselling unit*. *But ahhh…I won’t go there*. *I’m shy of the teachers*. *All they will do is to preach to you”!* ________*Female student*

*“When you go to the guidance and counselling*, *they will be talking plenty without providing anything [assistance] for you*, *so I won’t waste my time going to them*. *……*. *I will keep it to myself”*. ________Male student

It also came out that infrastructural deficit was a challenge, particularly lack of rooms designated solely for rendering counselling sessions. Also, students were unaware of the existence of the unit; those who did were oblivious of its function. Although both teachers and students found the unit commendable, they had reservations regarding safeguarding the confidentiality of patrons of the unit.

*“It’s about trust and in the school here*, *they know that information easily spreads*. *So if I tell you my problem*, *it will easily spread*. *Some of the teachers too have ‘okro’ mouths*, *they can’t keep anything to themselves”*. ________Housemistress

As a means of promoting students’ psychological wellbeing, the teachers suggested the introduction of mental health education into the secondary school curriculum. This they believe will contribute to prompt identification of abnormalities and curtail suicide tendencies.

*“just like we have this sex education thing*, *I think it [mental health education] should be added because of the way people are even committing suicide today because of some trivial issues”*. ________Class tutor

### Integration of findings

The triangulated findings are shown in Table 4. The qualitative inquiry revealed that the mental health difficulties encountered by the students were triggered by poverty, domestic violence, victimization, lack of psychosocial support, illicit drug use and issues with intimate relationships.

**Table 4:** Joint display of key quantitative and qualitative findings and implications for adolescents’ mental health care.

Triggers of mental health problems	Causes per qualitative findings	Effects per quantitative findings	Implications for adolescent mental health practice
Financial difficulty	Parents abandoning their responsibilities compelling students to engage in menial jobs to cater for themselves	↑ odds for peer problems (AOR:1.78 95% CI: 95% 1.03–3.08)	Although free SHS has been introduced, students from poor homes could be supported with their basic needs. Parents should be more responsible.
History of domestic violence	Complaints of maltreatment by step-parents	↑ odds for conduct problems (AOR:1.89 95% CI:1.08–3.28)	Public education on the effects of domestic violence on adolescents’ wellbeing and applying sanctions.
Bullying in school	Junior (lower grade) residential students bullied by their seniors	↑ odds for conduct (AOR: 2.32, 95% CI:1.23–4.369) and peer (CI:1.64 95% CI:1.04–2.58) problems	Stringent measures should be put in place to check bullying in schools. Culpable students should be reprimanded.
Lack of psychosocial support	Students do not disclose their mental health problems due to stigma and mistrust	↑ odds for emotional symptoms (AOR:3.39, 95% CI:1.31–8.79)	Sensitize parents to befriend their wards and be observant to detect when their wards have problems. Guidance and counselling units should be equipped with trained counsellors to support students.
Use of illicit drugs	Stimulants are used with the notion to increase learning hours and also forget one’s problems	↑ odds for emotional (AOR:6.86 95% CI:2.18–21.55) and hyperactivity problems (AOR: 5.54, 95% CI: 1.20–25.52)	Counselling on consequences of drug use, punishing illicit users and coaching on effective learning methods
Sexual relationships	“Heartbreaks” associated with intimate relationships with the opposite sex as well as financial hardships	↑ odds for emotional (AOR: 3.86, 95% CI:2.23–6.65) and peer problems (AOR: 3.00, 95% CI:2.00–4.50)	Schools and families should discourage adolescents from indulging in intimate relationships until mentally matured, and for latter to open-up when breakups occur, to receive the needed help.

## Discussion

Out of the 53.6% adolescents who experienced mental health difficulties, peer (20.5%), emotional (16.3%) and conduct (13.3%) problems were the most common. High prevalence of mental health problems has been reported among younger populations in sub-Sahara Africa [7, 22]. School-going adolescents, particularly females, are the most affected [5].

We observed that students who self-rated their academic performance as poor and those who received intense pressure from their parents to excel in academic work had a higher tendency for mental health challenges. After SHS, gaining admission into tertiary institutions in Ghana is highly competitive. Parents have high expectations of their wards and pressurize them to learn and excel academically. In India, and elsewhere, pressure from parents on their wards to excel in academic work is not uncommon [19]. Although some amount of positive pressure is needed, if not controlled, can impact negatively on adolescents’ mental health. This partly explains why secondary school students in Ghana have higher academic stress, depression and suicidal ideation than university students [29].

The influence of social factors on the mental health of adolescents cannot be over-emphasized [23, 38]. Adolescents from low socio-economic families are at a higher risk of experiencing mental health abnormalities than those from affluent families [21]. We confirmed this in this study where economic hardship emerged as a key predisposing factor. Parents of most of the students are informal sector workers, where income is generally meagre in Ghana. As observed from the qualitative inquiry, male students who encountered financial hardship did menial jobs to survive. In contrast, the females entered into sexual relationships with the hope to receive financial support and also use it as a medium to gain peer recognition and social acceptance. Breakups from such affairs when not appropriately managed leads to depression, self-harm and suicide [39, 40]. This explains why females have a higher likelihood of experiencing mental health challenges, especially emotional and peer problems. Other studies have observed similar findings and linked it to the high predisposition of females to depressive disorders [31, 41, 42].

As a means of mitigating the financial barrier to secondary education, the Government of Ghana in 2017 introduced the free SHS policy. Every child who qualifies for secondary education is electronically placed in an SHS and the fees absorbed by the government. Having abolished out-of-pocket fee payment, parents and students from poor backgrounds have some respite. Nonetheless, parents must be responsible for their wards’ upkeep as personal needs are not covered under the policy. The state could support students coming from impoverished homes with their basic personal effects.

In LMICs, mental illness is highly stigmatized [43]. Affected persons are mostly regarded as cursed, mad or possessed [44, 45] and thus often suffer in silence and isolation [46]. Because mental illness has spiritual connotations, those ‘afflicted’ seek divine intervention from all religious fonts [44–46]. Ghana is a religious society and superstition is an integral part of the belief system. Witchcraft and curses are often believed to cause not only mental illness [47] but general misfortunes in life. The belief that spiritual entities can influence mental illness makes indigenous and faith healing the first-line treatment option [47]. However, belief in supernatural causes of mental illness is not limited to Ghana alone but extends even to health professionals in industrialized countries like the United States, China and Brazil [48]. Although treatments meted to persons who seek spiritual intervention, for instance in prayer camps, are generally inhuman [49], religious support is known to influence hope, life satisfaction and emotional well-being [31] and could therefore be harnessed to promote mental health.

Again on the key findings, students who were bullied had two- to three-fold likelihood of experiencing mental health difficulties particularly conduct and peer problems. Bullying in schools is a common phenomenon worldwide [33, 50, 51]. Bullying behaviours have causal pathways with tobacco and illicit drug use and additionally, is associated with depression, anxiety, stress, altered sleep, loneliness and suicidal ideation [33, 50, 51]. We speculate that bullying victimization in schools could originate fundamentally from violence in the home or exposure to bullying at the basic (primary) level of education. The quantitative results show that students who experience family conflict are more likely to experience conduct problems portrayed through delinquent behaviours, bullying, alcohol, tobacco and substance use [20, 51]. Moreover, the common phenomenon where seniors (higher grade students) bully their juniors [50] leads us to conceptualize that bullying victimization could develop in a framework of experiential bullying. The bullying inclination resulting from having been previously bullied could be suppressed and later rekindled in the form of relayed bullying and reciprocal bullying.

We saw suicidal ideation emerging subtly in the qualitative interviews; an indication that it can escalate to suicidal behaviours if no action is taken. This is particularly so as suicide ideations (15.4%), threats (13.4%) and attempts (2.3%) are common among Ghanaian students. The trends are comparable to developed countries [52] and are thought to originate from family connections [53]. Tendencies for self-harm are often a result of less productive coping mechanisms [24–26]. Although banned in Ghana [54], use of stimulants and amphetamine such as tramadol and cannabis particularly marijuana to deal with stressors is common among a tenth of adolescents [32, 55, 56]. However, these drugs tend to impede judgement, wellbeing, academic performance and peer relationships [26, 56].

We observed an association of alcohol intake and suicidal thoughts with peer problems, and illicit drug use with emotional and hyperactivity problems. Peer problems, for instance, was more prevalent among males. Feelings of isolation, deprivation and hopelessness increase self-harm and suicidal tendencies [5, 10]. Interestingly, our findings revealed that receiving psychosocial support from friends increased by two to four folds the probability for mental health problems particularly conduct and hyperactivity when compared with family support. This goes to buttress the essence of eliciting support from credible sources.

Thus, having guidance and counselling units in SHS is a laudable innovation. However, we noted that its usefulness is derailed by concerns over trust, confidentially and calibre of the unit heads. As many adolescents can be reached in school, the school environment creates a unique opportunity to implement mental health interventions such as sensitization on preventive measures, early detection, tailored counselling and prompt referral for specialist attention. Creating awareness on the role of the guidance and counselling unit and employing trained counsellors to man the unit could enhance its usage and impact. Just as medical examination for physical health conditions is conducted before enrollment, mental health assessment could be a mandatory aspect of medical examination in schools.

If the school environment is to be mental health-friendly and efficient school-based support systems institutionalized, then fundamental lessons in mental health care are necessary. It could be incorporated into existing school curriculum as is done in Nicaragua [57]. In this regard, active collaboration from the Ghana Education Service, Mental Health Authority, parents and teachers’ association and religious bodies is pivotal. Mental health education should be introduced from the primary through to the university levels. It should be a non-examinable subject taught by trained personnel who have academic qualifications in psychology, mental health, psychiatry, guidance and counseling and related disciplines. The scope could cover common mental health problems among adolescents and youths including the signs and symptoms and triggers of mental health disorders, preventive measures, coping strategies and support services.

### Strengths and limitations

As we used a mixed-method approach and obtained perspectives from both students and teachers, it has enhanced the rigour of the study. We increased the sample size for the survey by 5% to account for non-response. Luckily, we had a 100% response rate thereby guaranteeing more reliable results with greater precision and statistical power. The qualitative inquiries enriched our understanding of the quantitative results as it provided explanations to most of the observations. Moreover, the quotes reported in this paper are verbatim as all interviews were conducted in English and no back translation was necessary. A key limitation is that only first and second-year students participated in the study. We could not capture the views of third-year students as they were not present on campus during the data collection. Also, the study was restricted to only school-going adolescents. The results cannot be generalized to non-school-going adolescents as the school setting triggers peculiar mental health problems. Therefore, similar studies could be replicated among non-school-going adolescents. Also, one might consider exploring how the guidance and counseling unit could be strengthened and also investigate the ability of the unit in tackling the mental health needs of students.

## Conclusion

Since adolescents spend most of their time in school, making the school environment mental health-friendly is fundamental in promoting psychological well-being. We suggest strengthening guidance and counselling units and training qualified personnel who understand the tenets of psychotherapy to provide the service. Finally, basic education in mental health is essential and need to be incorporated into the existing school curriculum.

## Supporting information

S1 File (SAV)
